# Supplementary material for: Application of Bacteriophages on Shiga Toxin-Producing Escherichia coli (STEC) Biofilm
Source: Antibiotics (Basel). 2021 Nov 20;10(11):1423. doi: 10.3390/antibiotics10111423 (PMC8614735; doi:10.3390/antibiotics10111423)
Supplement: Supplementary file 1 [file antibiotics-10-01423-s001.zip › antibiotics-1424365-supplementary.pdf]

antibiotics

MDPI

Supplementary Table S1. Average of OD value. $^{+}$Dev St is for standard deviation. *CONTROL: refers to the bacterial strain cultivation without any phage addition. **% of reduction: calculated as the overall MOI average out of each bacterial control.

		ATCC 25404	Dev St^{+}	CNCTC 6246	Dev St^{+}	C679-12	Dev St^{+}	ED56	Dev St^{+}	ED33	Dev St^{+}	ED226	Dev St^{+}
MOI 1	LF2	0.146	0.014	0.127	0.003	0.222	0.018	0.223	0.048	0.177	0.028	0.228	0.02
	FM9	0.144	0.005	0.125	0.009	0.188	0.01	0.233	0.025	0.185	0.014	0.199	0.017
	FM10	0.147	0.011	0.121	0.002	0.113	0.005	0.189	0.023	0.197	0.013	0.186	0.013
	DP16	0.157	0.011	0.124	0.002	0.177	0.015	0.19	0.009	0.164	0.018	0.144	0.011
	DP17	0.139	0.01	0.124	0.002	0.165	0.019	0.235	0.061	0.178	0.017	0.159	0.026
	DP19	0.159	0.015	0.13	0.007	0.15	0.006	0.194	0.01	0.197	0.034	0.168	0.026
	Cocktail 1	0.149	0.004	0.125	0.005	0.133	0.007	0.182	0.006	0.178	0.011	0.131	0.016
	Cocktail 2	0.14	0.006	0.131	0.007	0.146	0.013	0.199	0.019	0.192	0.015	0.148	0.011
MOI 2	LF2	0.164	0.005	0.15	0.058	0.292	0.022	0.283	0.028	0.207	0.014	0.258	0.005
	FM9	0.161	0.007	0.12	0.004	0.341	0.011	0.29	0.031	0.186	0.009	0.193	0.009
	FM10	0.157	0.02	0.133	0.015	0.285	0.006	0.231	0.023	0.246	0.016	0.186	0.04
	DP16	0.138	0.006	0.121	0.004	0.287	0.016	0.258	0.013	0.193	0.022	0.233	0.017
	DP17	0.125	0.003	0.118	0.004	0.262	0.005	0.276	0.018	0.192	0.004	0.161	0.007
	DP19	0.161	0.007	0.149	0.006	0.286	0.01	0.218	0.012	0.2	0.02	0.27	0.023
	Cocktail 1	0.155	0.01	0.131	0.01	0.296	0.016	0.234	0.02	0.183	0.004	0.291	0.017
	Cocktail 2	0.125	0.005	0.129	0.001	0.298	0.037	0.231	0.006	0.169	0.008	0.126	0.01
MOI 10	LF2	0.148	0.023	0.13	0.007	0.269	0.032	0.188	0.018	0.187	0.015	0.164	0.003
	FM9	0.121	0.003	0.129	0.007	0.284	0.058	0.185	0.012	0.187	0.011	0.166	0.006
	FM10	0.139	0.015	0.123	0.008	0.145	0.039	0.158	0.012	0.213	0.023	0.204	0.016
	DP16	0.126	0.008	0.114	0.002	0.329	0.013	0.214	0.005	0.183	0.003	0.194	0.026
	DP17	0.122	0.005	0.114	0.001	0.212	0.076	0.191	0.029	0.175	0.009	0.169	0.006
	DP19	0.152	0.017	0.117	0.003	0.29	0.068	0.159	0.004	0.18	0.016	0.178	0.027
	Cocktail 1	0.12	0.005	0.118	0.002	0.128	0.002	0.169	0.017	0.184	0.008	0.13	0.013
	Cocktail 2	0.12	0.001	0.12	0.004	0.134	0.006	0.178	0.001	0.178	0.01	0.127	0.002
MOI 100	LF2	0.158	0.005	0.124	0.004	0.149	0.031	0.199	0.01	0.162	0.007	0.187	0.011
	FM9	0.129	0.008	0.11	0.003	0.126	0.022	0.218	0.01	0.162	0.004	0.176	0.009
	FM10	0.137	0.012	0.117	0.008	0.122	0.007	0.167	0.005	0.151	0.005	0.192	0.008
	DP16	0.131	0.002	0.112	0.013	0.117	0.005	0.185	0.013	0.152	0.008	0.187	0.025
	DP17	0.125	0.004	0.119	0.002	0.136	0.033	0.193	0.022	0.157	0.011	0.189	0.002
	DP19	0.133	0.024	0.125	0.001	0.139	0.004	0.168	0.009	0.162	0.005	0.196	0.024
	Cocktail 1	0.127	0.013	0.119	0.005	0.126	0.006	0.171	0.005	0.161	0.005	0.168	0.013
	Cocktail 2	0.127	0.002	0.127	0.009	0.136	0.005	0.172	0.009	0.163	0.009	0.158	0.018
	Average	0.139	0.007	0.124	0.004	0.171	0.013	0.1935	0.0125	0.1815	0.011	0.182	0.013
	CONTROL*	0.287	0.079	0.388	0.019	0.334	0.072	0.134	0.003	0.443	0.031	0.24	0.025
	% of reduction**	51.57		68.04		48.80		-44.40		59.03		24.17	
